# The interaction between artemether-lumefantrine and lopinavir/ritonavir-based antiretroviral therapy in HIV-1 infected patients

**DOI:** 10.1186/s12879-016-1345-1

**Published:** 2016-01-27

**Authors:** T. Kredo, K. Mauff, L. Workman, J. S. Van der Walt, L. Wiesner, P. J. Smith, G. Maartens, K. Cohen, K. I. Barnes

**Affiliations:** 1 Division of Clinical Pharmacology, Department of Medicine, University of Cape Town, Cape Town, South Africa; 2 Cochrane South Africa, South African Medical Research Council, Cape Town, South Africa; 3 Department of Statistical Sciences, University of Cape Town, Cape Town, South Africa; 4 WorlldWide Antimalarial Resistance Network (WWARN), Oxford, UK

**Keywords:** HIV, Malaria, Artemether, Lumefantrine, Lopinavir, Ritonavir, Drug interaction, Safety, Pharmacokinetic, Dose-related exposure

## Abstract

**Background:**

Artemether-lumefantrine is currently the most widely recommended treatment of uncomplicated malaria. Lopinavir–based antiretroviral therapy is the commonly recommended second-line HIV treatment. Artemether and lumefantrine are metabolised by cytochrome P450 isoenzyme CYP3A4, which lopinavir/ritonavir inhibits, potentially causing clinically important drug-drug interactions.

**Methods:**

An adaptive, parallel-design safety and pharmacokinetic study was conducted in HIV-infected (malaria-negative) patients: antiretroviral-naïve and those stable on lopinavir/ritonavir-based antiretrovirals. Both groups received the recommended six-dose artemether-lumefantrine treatment. The primary outcome was day-7 lumefantrine concentrations, as these correlate with antimalarial efficacy. Adverse events were solicited throughout the study, recording the onset, duration, severity, and relationship to artemether-lumefantrine.

**Results:**

We enrolled 34 patients. Median day-7 lumefantrine concentrations were almost 10-fold higher in the lopinavir than the antiretroviral-naïve group [3170 versus 336 ng/mL; *p* = 0.0001], with AUC_(0-inf)_ and C_max_ increased five-fold [2478 versus 445 μg.h/mL; *p* = 0.0001], and three-fold [28.2 versus 8.8 μg/mL; p < 0.0001], respectively. Lumefantrine C_max,_ and AUC_(0-inf)_ increased significantly with mg/kg dose in the lopinavir, but not the antiretroviral-naïve group. While artemether exposure was similar between groups, C_max_ and AUC_(0-8h)_ of its active metabolite dihydroartemisinin were initially two-fold higher in the lopinavir group [*p* = 0.004 and *p* = 0.0013, respectively]. However, this difference was no longer apparent after the last artemether-lumefantrine dose. Within 21 days of starting artemether-lumefantrine there were similar numbers of treatment emergent adverse events (42 vs. 35) and adverse reactions (12 vs. 15, *p* = 0.21) in the lopinavir and antiretroviral-naïve groups, respectively. There were no serious adverse events and no difference in electrocardiographic QTcF- and PR-intervals, at the predicted lumefantrine T_max_.

**Conclusion:**

Despite substantially higher lumefantrine exposure, intensive monitoring in our relatively small study raised no safety concerns in HIV-infected patients stable on lopinavir-based antiretroviral therapy given the recommended artemether-lumefantrine dosage. Increased day-7 lumefantrine concentrations have been shown previously to reduce the risk of malaria treatment failure, but further evidence in adult patients co-infected with malaria and HIV is needed to assess the artemether-lumefantrine risk : benefit profile in this vulnerable population fully. Our antiretroviral-naïve patients confirmed previous findings that lumefantrine absorption is almost saturated at currently recommended doses, but this dose-limited absorption was overcome in the lopinavir group.

**Trial registration:**

Clinical Trial Registration number NCT00869700. Registered on clinicaltrials.gov 25 March 2009

**Electronic supplementary material:**

The online version of this article (doi:10.1186/s12879-016-1345-1) contains supplementary material, which is available to authorized users.

## Background

With the overlapping geographic distribution of HIV and *P. falciparum* malaria, many patients may require co-treatment with antiretrovirals and antimalarials. For uncomplicated malaria, the World Health Organization (WHO) recommends artemisinin-based combination therapies (ACTs), of which the fixed-dose combination artemether-lumefantrine is currently most widely used, accounting for 73 % of ACTs procured in 2013 [1]. The precise pharmacokinetic determinants of treatment outcome in uncomplicated malaria remain uncertain, but the area under the concentration-time curve (AUC) and the concentration on day-7 of slowly eliminated antimalarials are considered important predictors [2, 3]. The ‘therapeutic’ day-7 lumefantrine concentrations published to date range between 170 ng/mL to 500 ng/mL, with a concentration of 280 ng/mL most often cited [4–12].

As the HIV pandemic matures, increasing numbers of patients develop resistance to first-line antiretroviral therapy (ART) and are placed on second-line ART. Ritonavir-boosted lopinavir-based ART is the most widely used second-line ART in Africa and South East Asia. Artemether, lumefantrine and lopinavir are all primarily metabolised by the same cytochrome P450 (CYP) iso-enzyme, CYP3A4. Ritonavir is a potent inhibitor of CYP3A4 creating the potential for clinically significant drug-drug interactions [13]. Although the interaction between lopinavir and ritonavir is used for therapeutic advantage, known as ‘boosting’, there is limited evidence to inform clinicians and policy makers about the interaction between artemether-lumefantrine and lopinavir-based ART, leading to inconsistent recommendations on the use of artemether-lumefantrine in patients co-infected with HIV/AIDS [12, 14, 15]. As access to antiretrovirals and ACTs increase, the importance of defining the interaction between antimalarials and ART becomes more urgent. Our study investigated the pharmacokinetics and safety of the recommended adult dose of artemether-lumefantrine when given to HIV-infected patients stable on lopinavir-based ART.

## Methods

### Subjects and study design

We conducted a sequential, two-period, adaptive design, open-label, pharmacokinetic and safety drug-drug interaction study at the Groote Schuur Hospital Clinical Pharmacology Research Ward in Cape Town, South Africa.

HIV-infected adults (18 years of age or older) with CD4+ lymphocyte counts greater than 200 cells/μL were enrolled. Participants enrolled were stable on treatment with lopinavir-based ART for a minimum of six weeks. They were compared with a group of patients who were antiretroviral (ARV)-naïve and not yet eligible for ART, according to the South African National HIV Treatment Guidelines at the time [16, 17]. The participants were otherwise well adults without renal disease and were not geriatric, underweight, overweight or obese [18].

Exclusion criteria for safety reasons were a current diagnosis of malaria, known hypersensitivity to artemether or lumefantrine, pregnancy (as confirmed by a serum Beta-HCG test), breast-feeding, or clinically relevant hepatic or renal dysfunction. In addition, those with a pre-existing (or family history of) prolonged QT interval, cardiac dysrhythmia, electrolyte disturbances or taking any drugs known to prolong the QT interval, were excluded. Exclusion criteria for potential confounding of the pharmacokinetic parameters included participants using other drugs known to interact via the CYP450 enzyme system, current smokers, or alcohol users who would not abstain from alcohol intake for the trial duration. Caffeine, grapefruit juice or strenuous exercises were not permitted from 24 h before and during study admission.

### Ethics, consent and permissions

Patients provided written informed consent prior to enrollment. Regulatory approval was received from the University of Cape Town Research Ethics Committee and the South African Medicines Control Council (Clinical Trial Registration number NCT00869700). The procedures followed were in accordance with the Good Clinical Practice Guidelines, including the Helsinki Declaration of 1975, as revised in 2008.

### Dosing and pharmacokinetic sample collection

As there was a safety concern about increases in lumefantrine concentrations secondary to inhibition by ritonavir and lopinavir, patients on lopinavir-based treatment were admitted for a single dose of artemether-lumefantrine (80 mg/480 mg) in a dose-finding safety phase. Pharmacokinetic and safety results were analysed and reviewed by the Data Safety Monitoring Board prior to approval of the adapted dose used in the multiple-dosing phase. The ARV-naïve participants took part in the multiple-dosing phase only, when the recommended adult 80 mg/480 mg artemether-lumefantrine dose was given at 0, 8, 24, 36, 48 and 60 h [12] .

In both study groups, all doses were administered with 40 mL of soya milk (0.8 g fat) and a meal containing a minimum of 6 g of fat within one hour of each dose, with the exception of dose 2 (at 8 h) when only soya milk accompanied the dose.

Participants were admitted for rich pharmacokinetic sampling (until 72 h after the first artemether-lumefantrine dose). Subsequent samples were collected on an outpatient basis until day 21. Venous blood samples were collected into heparinised (LH PST II) BD Vacutainer® tubes. The blood tubes were pre-chilled on ice for 10 min; all samples were again chilled before being centrifuged at 4 °C for 10 min at 2000 g. The resulting plasma was stored at −80 °C within 30 min of the blood draw. Pharmacokinetic assays were done within four months of sample collection.

For the Phase 1 (single-dose) pharmacokinetic profile: Plasma concentrations of lumefantrine were assayed at pre-dose (0 h), 0.5,1, 1.5, 2, 3, 4, 5, 6, 8, 14, 24, 36, 48, 60, 72, 96, 120, 144, 168, 336 and 504 h post and artemether/dihydroartemisinin concentrations were assayed at pre-dose (0 h), 0.5, 1, 1.5, 2, 3, 4, 5, 6, 8, and 24 h after the first artemether-lumefantrine dose.

For the Phase 2 (full-treatment dose) pharmacokinetic profile: Plasma concentrations of lumefantrine were assayed at pre-dose (0 h), 0.5,1, 1.5, 2, 3, 4, 5, 6, 8, 14, 24, 30, 36, 42, 48, 54, 60, 61.5, 62, 63, 64, 65, 66, 68, 70, 72, 96, 120, 144, 168, 336 and 504 h, and artemether/dihydroartemisinin concentrations were assayed at pre-dose (0 h), 0.5, 1, 1.5, 2, 3, 4, 5, 6, 8, 24, 60, 61.5, 62, 63, 64, 65, 66, 68, 70 and 72 h post-dose.

### Pharmacokinetic assays

Concentrations of lumefantrine, artemether, and dihydroartemisinin were determined by the Division of Clinical Pharmacology Laboratory, University of Cape Town using validated liquid chromatography tandem mass spectrometer (LC-MS/MS) assays as described previously [16].

### Safety data collection

A clinical evaluation and full-blood count, renal function tests, liver enzymes, lactate and glucose blood tests were performed at screening and at the final safety visit 21 days after the first artemether-lumefantrine dose in both the single-dose and multiple-dose phases of the study. CD4+ lymphocyte counts and HIV-1 viral loads and serum pregnancy tests (in all women) as well as urine tests for drugs of abuse (amphetamines, benzodiazepines and opiates) were performed at screening. Twelve-lead single electrocardiograms (ECGs) were performed at screening, pre-dose and at the expected time of maximal lumefantrine plasma concentration (68 h post-dose) [19]. An independent cardiologist assessed all ECGs and the QT interval was corrected using the Fridericia formula [20]. Adverse events were solicited throughout the study, starting on completion of screening and recording the onset, duration, severity, relationship to study drug and need for treatment [21, 22]. These were classified using MedDRA preferred terms. Some participants in the lopinavir group were also included in a methods sub-study evaluating more intensive methods for eliciting adverse event data from participants including checklists, in-depth interviews and focus group discussions [22].

### Statistical methods

The sample size was calculated to demonstrate a 2-fold change in lumefantrine exposure (day 7 concentration or AUC), i.e., such that the 90 % confidence intervals (CIs) for geometric mean ratios lie outside the interval 0.5 to 2.0 with a power of 80 % [19]. Thirteen participants were required in each group and a total of 18 participants were recruited for each arm to accommodate potential dropouts [16].

Data analysis and pharmacokinetic modelling (non-compartmental) were performed using Stata 13 (StataCorp, College Station, Texas). Concentrations below the limits of quantification were considered missing. Area under the concentration-time curve (AUC_0-∞_) was calculated using the trapezoidal rule. Elimination half-life was calculated as ln(2) ⁄ λz, where λz is the first order rate constant associated with the terminal (log-linear) portion of the curve, estimated by linear regression of time vs. log concentration, using the default of last three data points.

In order to predict a safe dose for administration in Phase 2, the lumefantrine concentration-time data (0–8 h) from our single-dose safety phase (Phase 1) were compared with those in 18 ARV-naïve subjects included in our prior antimalarial-antiretroviral drug interaction study using geometric mean ratios [16]. In the latter study the subjects completed a full course of artemether-lumefantrine using the same schedule as in the multiple-dose phase.

Determinants of lumefantrine day-7 concentrations, AUC_(0-inf)_ and C_max_ values were explored using linear regression of the log transformed values, with results reported as geometric mean ratios (GMR). The Spearman rank correlation test was used to test the correlation between lumefantrine day-7 concentrations and AUC_(0-inf)_. Logistic regression was used to explore the determinants of day-7 lumefantrine concentrations below the reported therapeutic concentration (280 ng/mL). Continuous and categorical covariates were compared between groups at baseline using Kruskal-Wallis and Chi-squared tests, respectively. Kruskall Wallis tests were also used for simple comparisons of the day-7 lumefantrine concentrations, AUC and C_max_ values between groups. In order to account for the repeated measures per subject, particularly given previously reported auto-induction effects with the artemisinins, mixed-effect regression models were used to assess the possible impact of dose-occasion on artemether and dihydroartemisinin exposure, where the responses were log-transformed AUC and C_max_ values.

Secondary safety endpoints included frequency and severity of adverse events, changes in haematological, serum biochemical and urinalysis parameters, and vital signs between screening and follow-up. The risk of adverse drug reactions was compared between treatment groups using logistic regression. ECG parameters (PR-, QRS-, RR- and QT-intervals) were compared within groups between screening and the predicted lumefantrine T_max_ using the Wilcoxon signed rank test, while the Wilcoxon rank sum test was used to compare these between groups and within period, and their correlation with lumefantrine concentrations was assessed using the Spearman Rank correlation test.

## Results

### Study population

Thirty-six adults (18 in the ARV-naïve group and 18 in the lopinavir group) were recruited. All 18 in the ARV-naïve group and 16/18 (89 %) in the lopinavir group completed the study. One participant was replaced after being excluded prior to Phase 2 dosing due to starting a potentially interacting medication (amitriptyline); two participants withdrew consent during phase 2 (one to attend a funeral in another province, the other provided no explanation). At baseline, the groups were well matched for weight-adjusted (mg/kg) lumefantrine dose and CD4+ lymphocyte count. ARV-naïve patients were younger (*p* = 0.0001), and had a higher median viral load (3.76 log_10_ copies/mL; *p* = 0.0005). The mean corpuscular volume was higher with ARV use, as is expected with AZT and d4T. Gamma glutamyl-transferase levels were higher in the lopinavir group than the ARV-naïve group (median 26 (IQR 18–44) U/L vs. 15 (13–29) U/L; *p* = 0.03), which reflects the higher upper limit of the normal range in males than females (60 vs. 35 U/L); males made up 6 % of the ARV-naïve group and 31 % of the lopinavir group. Six participants in the ARV-naïve group and five in the lopinavir group (*p* = 1.0) were receiving cotrimoxazole prophylaxis (Table 1).

**Table 1:** Baseline characteristics in HIV-1 infected patients who are antiretroviral-naïve (*n* = 18) or on lopinavir-based antiretroviral therapy (*n* = 16)

Parameter	ARV-Naïve group	Lopinavir group	*p* value
Sex female n (%)	17 (94 %)	11 (69 %)	0.078
Age (years)	27 (25–32)	37 (33–41)	<0.0001
Total lumefantrine dose (mg/kg)	49.7 (43.0–52.4)	46.5 (41.4–51.9)	0.72
Weight (kg)	58 (55–67)	62 (56–70)	0.72
Albumin (g/L)	40 (39–44)	43 (41.5–44)	0.14
Alkaline phosphatase (U/L)	57.5 (50–69)	69.5 (59–88)	0.09
Gamma glutamyltransferase (U/L)	40 (39–44)	43 (42–44)	0.03
Alanine transaminase (U/L)	15 (13–29)	26 (18–44)	0.72
Aspartate transaminase (U/L)	22 (14–28)	19 (17–30)	0.59
Mean corpuscular volume (fL)	86.7 (85–91)	100.95 (98–110)	0.0001
Concomitant cotrimoxazole n (%)	6 (33 %)	5 (31 %)	1.0
CD4+ count (×10^6^/L)	356 (260–507)	375 (296–590)	0.7
HIV viral load (copies/mL)	log_10_ 3.76 (3.0–4.1)	<50 copies/μL	0.0005

Values are shown as medians (interquartile ranges [IQRs]) or n (%). Statistical significance (p values) calculated using the Kruskal-Wallis test or chi-squared test, as appropriate

### Pharmacokinetic results

#### Single-dose safety phase (Phase 1)

The non-compartmental analysis of the single artemether-lumefantrine dose, safety phase (Phase 1) in the lopinavir group was compared with the ARV-naïve group. The GMR (90 % CI) for the lumefantrine C_max_ was 1.86 (1.48–2.33), while that for the lumefantrine AUC_(0-8h)_ was 1.78 (1.43–2.33). The Data Safety Monitoring Board and investigators agreed based on the predefined criteria (i.e., GMR between 0.5 and 2) to continuing to Phase 2 using the full recommended adult six-dose artemether-lumefantrine regimen.

#### Effect of lopinavir-based ART on lumefantrine plasma concentrations following six-dose artemether-lumefantrine regimen (Phase 2)

**Fig. 1:**
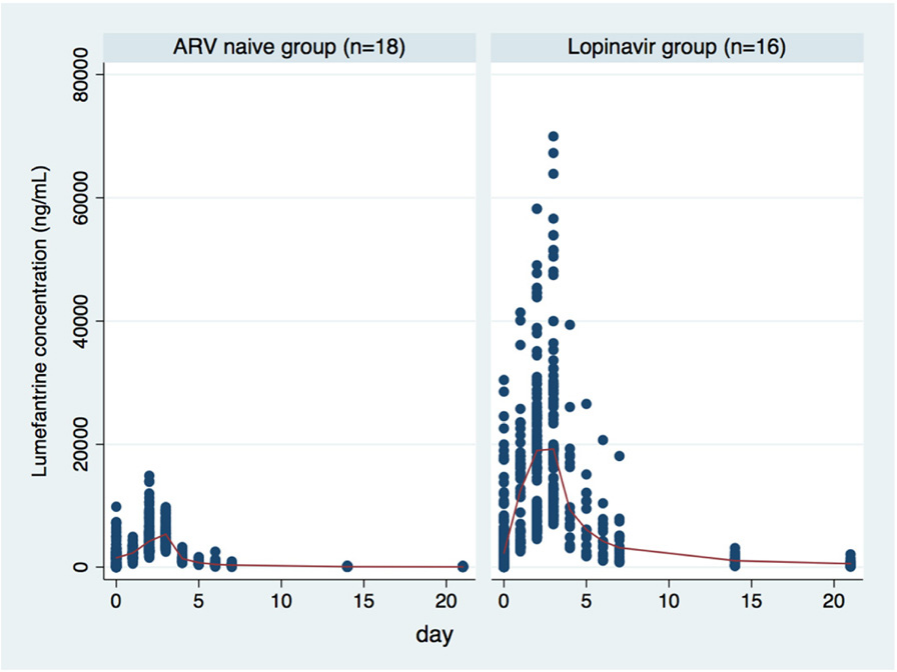
Scatter plot of Plasma lumefantrine concentrations over time, by study group

The lumefantrine plasma concentration-time curves (0–504 hours) are depicted in Fig. 1 and summarised in Table 2. Median lumefantrine maximum (C_max_) concentrations of 8.76 and 28.15 μg/mL were achieved at a median time (T_max_) of 42 and 67 h respectively in the ARV-naïve and lopinavir groups. The median area under the plasma lumefantrine concentration time curve (AUC_(0-inf)_) was 2478 μg.h/mL in the lopinavir group and 445 μg.h/mL in the ARV-naïve group (*p* = 0.0001). Elimination half-life also appeared longer in the lopinavir group (4.6 vs 4.1 h, *p* = 0.0027), although this is more likely to reflect improved bioavailability with lopinavir co-administration.

**Table 2:** Lumefantrine pharmacokinetic parameters following six-dose artemether-lumefantrine treatment in HIV-1 infected patients who are antiretroviral-naïve or on lopinavir-based antiretroviral therapy

Parameter median (IQR)	ARV-naïve group (*n* = 18)	Lopinavir group (*n* = 16)	*p* value
C_max_ (μg/mL)	8.76 (7.80–9.84)	28.15 (14.00–32.95)	<0.0001
CV	26.3 %	59.6 %	
T_max_ (h)	42 (42–66)	67 (51–70)	0.031
CV	35.5 %	27.1 %	
AUC_(0-inf)_ (μg.h/mL)	445 (357–553)	2478 (1093–3596)	<0.0001
CV	33.5 %	71.0 %	
T_1/2_ (days)	4.1 (2.7–4.4)	4.6 (4.4–5.2)	0.003
CV	25.7 %	15.4 %	
Day-7 conc (ng/mL)	336 (230–396)	3170 (1440–5085)	<0.0001
CV	54.0 %	98.5 %	

Values are shown as medians (interquartile ranges [IQRs]) and Coefficient of variation (CV, %). *C*max, maximal concentration; *T*max, time at the maximal concentration; AUC(0-inf), area under the plasma concentration-time curve, from 0 h to infinity; *t*1/2, elimination half-life; Day-7 concentration, lumefantrine concentration on day-7;. Statistical significance (p values) calculated using the Kruskal-Wallis test

Median (range) day-7 lumefantrine concentrations were 3170 (772–18,100) ng/mL in the lopinavir group compared to 336 (29–934) ng/mL in the ARV-naïve group (*p* = 0.0001). Across both groups, each 1 mg/kg increase in the lumefantrine dose increased day-7 lumefantrine concentrations by 5.9 % (95 % CI 2.1–9.8 %; *p* = 0.003). After adjusting for mg/kg dose, the lumefantrine day-7 concentration for the lopinavir group was 10-fold those in the ARV-naïve group (adjusted GMR 10.4 [95 % CI 6.4–16.9]; *p* < 0.0001). None of the subjects in the lopinavir group had day-7 lumefantrine concentrations below a therapeutic threshold (280 ng/mL), compared to one-third (6/18) of those in the ARV-naïve group (*p* = 0.02). Lumefantrine day-7 concentrations and AUC_(0-inf)_ were highly correlated (R-squared = 0.98).

**Fig. 2:**
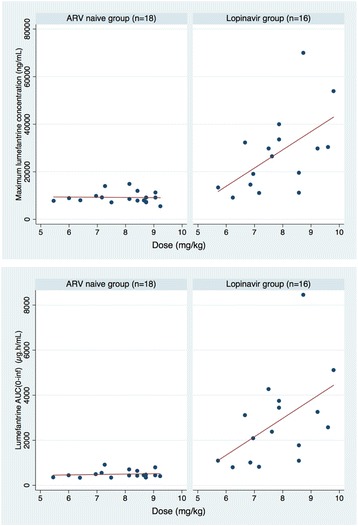
Scatter plot showing the effect of mg/kg lumefantrine dose (given twice daily for three days) on **a** lumefantrine maximum concentration (upper panel) and **b** lumefantrine area under the concentration time curve (AUC(0-inf)) (lower panel), by treatment group

Adjusting for mg/kg dose (the only significant covariate), the median lumefantrine AUC_(0-inf)_ was almost five-fold higher (GMR 4.82 95 % CI 3.41–6.79, *p* < 0.0001) and the median C_max_ values more than doubled (GMR 2.67 95 % CI 1.99–3.59; *p* < 0.0001) in the lopinavir than the ARV-naïve group. However, the increase in exposure with mg/kg dose was only seen in the lopinavir group in whom the C_max_ increased by 4.8 % (95%CI 0.9–8.6 %; *p* = 0.019) and the AUC_(0-inf)_ increased by 5.4 % (95%CI 0.7–10.0 %; *p* = 0.026), respectively with each 1 mg/kg increase in lumefantrine dose (Fig. 2).

### Effect of lopinavir-based ART on artemether and dihydroartemisinin plasma concentrations following six-dose artemether-lumefantrine regimen (Phase 2)

**Fig. 3:**
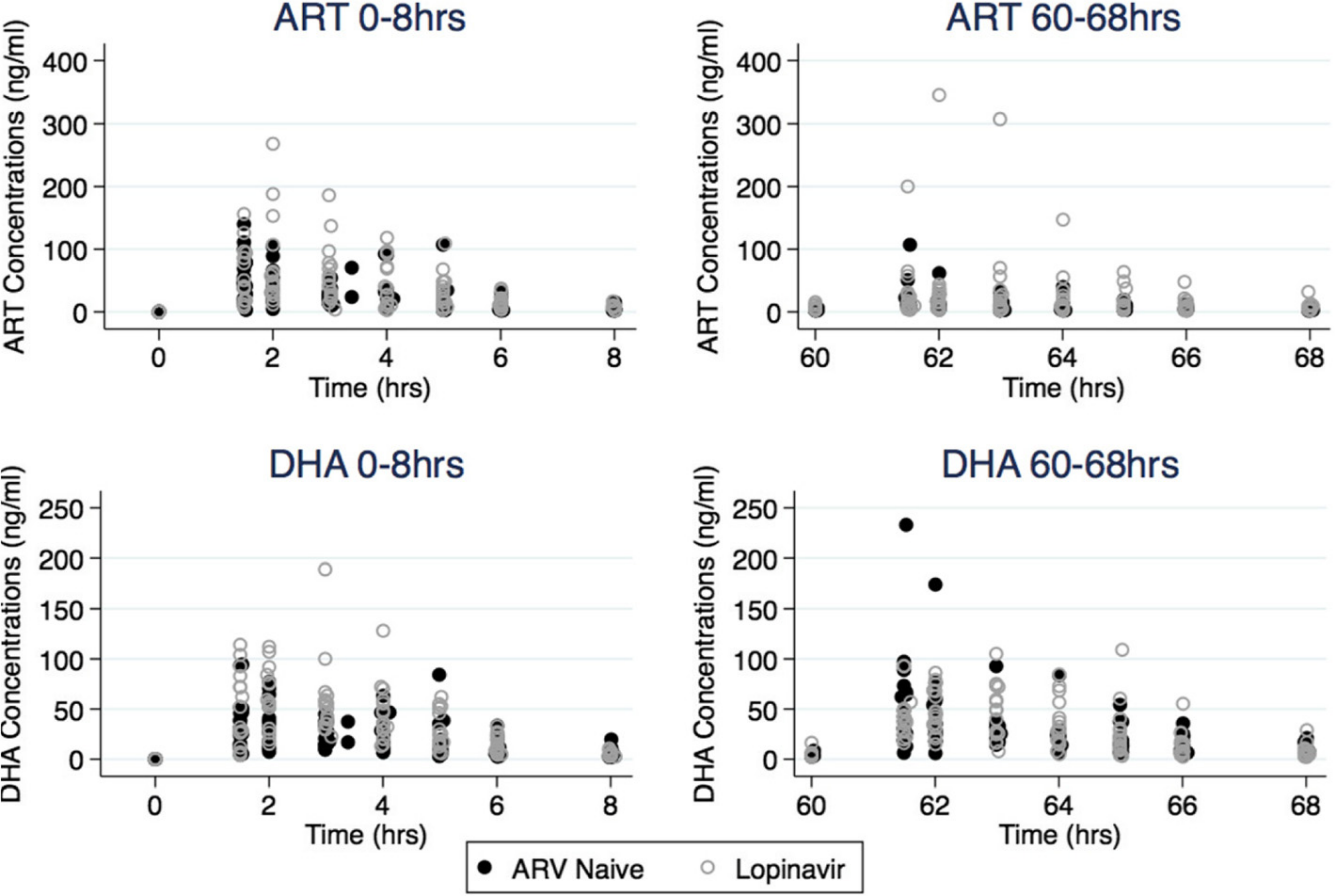
Scatter plot of Plasma artemether (ART) and dihydroartemisinin (DHA) concentrations over time, by study group and treatment period (after dose 1 (0–8 hours), and after dose 6 (60–68 hours))

The artemether and dihydroartemisinin plasma concentration-time curves after artemether-lumefantrine dose one (0–8 hours) and dose six (60–68 hours) are depicted in Fig. 3, with artemether and dihydroartemisinin pharmacokinetic parameters summarised by treatment group in Table 3.

**Table 3:** Artemether and dihydroartemisinin pharmacokinetic parameters in HIV-1 infected patients who are antiretroviral (ARV)-naïve or on lopinavir-based antiretroviral therapy (ART), after artemether-lumefantrine dose 1 (0–8 hours) and dose 6 (60–68 hours)

0–8 hours				60–68 hours		
	ARV-Naïve group	Lopinavir group	*p* value	ARV-Naïve group	Lopinavir group	*p* value
Artemether						
C_max_ (ng/mL)	59.7 (37.8–88.9)	85.8 (39.7–145)	0. 16	11.9 (8.2–17.5)	16.5 (7.2–50.5)	0.35
T_max_ (h)	1.5 (1.5–2.0)	1.8 (1.5–3.0)	0. 97	61.5 (61.5–62.0)	61.5 (61.5–62.0)	0. 84
AUC_(0-inf)_ (ng.h/mL)	151.0 (110.7–220.6)	220.0 (113.9–431.2)	0.17	71.1 (45.5–114.2)	93.8 (37.5–219.1)	0. 37
t_1/2_ (hr)	1.5 (1.1–1.7)	1.4 (0.9–1.6)	0. 39	2.9 (1.8–5.4)	3.0 (1.9–3.4)	0. 68
Dihydroartemisinin						
C_max_ (ng/mL)	42.2 (31.8–63.1)	77.5 (59.4–102)	0.004	40.0 (31.2–66.7)	65.8 (38.2–92.7)	0.21
T_max_ (h)	2.0 (1.5–4.0)	2.0 (1.5–3.0)	0.41	61.5 (61.5–62.0)	61.6 (61.5–62.5)	0.71
AUC_(0-inf)_ (ng.h/mL)	123.8 (101.3–235.6)	283.6 (178.1–340.7)	0.001	165.7 (143.7–246.5)	243.5 (145.1–305.0)	0.27
T_1/2_ (h)	1.6 (1.3–2.1)	1.4 (1.1–1.6)	0. 07	2.0 (1.8–2.7)	1.8 (1.5–2.0)	0. 02

Values are reported as median (interquartile range [IQR]). *C*max, maximal concentration; *T*max, time at the maximal concentration; AUC (0-inf), area under the plasma concentration-time curve, from 0 h to infinity; *t*1/2, elimination half-life. Statistical significance (p values) calculated using the Kruskal-Wallis test

For artemether, there were no significant differences between treatment groups for any of the pharmacokinetic parameters at either of the time periods studied, 0 to 8 h and 60 to 68 h (Table 3). However, the mixed-effect model showed a significant dose-occasion effect on the artemether AUC and C_max_ in both treatment groups. After the last artemether-lumefantrine dose (60–68 hours), artemether C_max_ was 76 % lower (GMR 0.24 [95 % CI 0.17–0.34]) and AUC was 58 % lower (GMR 0.42 [95 % CI 0.30–0.59]; *p* < 0.0001) than after the first artemether-lumefantrine dose.

**Fig. 4:**
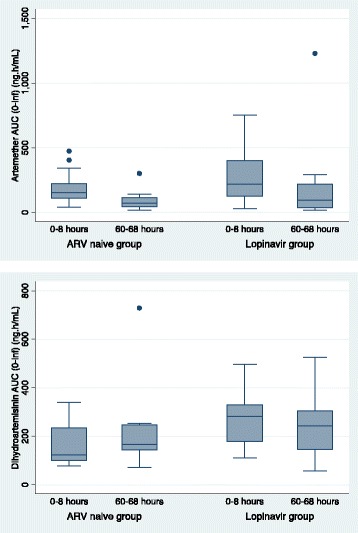
Box plot of area under the plasma artemether (ART, upper panel) and dihydroartemisinin (DHA, lower panel) concentration time curves (0-infinity) ng.h/mL after artemether-lumefantrine dose 1 (0–8 hours) and dose 6 (60–68 hours), by study group

For dihydroartemisinin, after dose 1 (0 to 8 h), the lopinavir group had almost double the exposure of the ARV-naïve group (median [range] C_max_ of 77.5 [30.3–189.0] vs. 42.2 [17.4–94.4] ng/mL; *p* = 0.004) and AUC_(0-8h)_ of 283.6 [110.1–495.6] vs. 123.8 [79.7–340.0] ng.h/mL; *p* = 0.001). By 60 to 68 h (after dose 6), exposure was similar between treatment groups other than slight differences in the elimination half-life (Table 3, Figs. 3 and 4). These findings were confirmed in the mixed-effect model, which also showed that the dihydroartemisinin AUC increased significantly between the first and last dose in the ARV-naïve group (GMR 1.30 [95 % CI 1.03–1.64], *p* = 0.03) but not in the lopinavir group (*p* = 0.195) (Table 4).

**Table 4:** Mixed-effects model of the effects of dose occasion, treatment group and covariates on dihydroartemisinin exposure in HIV-1 infected patients

Dose occasion effect (last/first dose occasion)	AUC GMR (95 % CI)	C_max_ GMR (95 % CI)
- Antiretroviral naïve group	1.30 (1.03–1.64)	1.12 (0.84–1.49)
- Lopinavir group	0.84 (0.65–1.09)	0.77 (0.56–1.05)
Treatment group effect (lopinavir group/naïve group)		
- 0 to 8 h	1.73 (1.26–2.37)	1.73 (1.24–2.43)
- 60 to 68 h	1.12 (0.56–1.55)	1.19 (0.58–2.57)

### Electrocardiograph safety

The QTcF intervals were similar between treatment groups at the predicted time to maximum concentration (~Tmax, 68 h after the first dose, which was very close to the observed Cmax of 67 h in the lopinavir group) and within groups between screening and ~ Tmax. There were no QTcF intervals > 450 msec, even at ~ Tmax. The median (range) QTcF intervals were 406 (370–443) ms at ~ Tmax in the lopinavir group, compared with 409 (366–436) ms in the ARV-naïve group (*p* = 0.72) at 68 h, and 408 (370–438) ms in the lopinavir group at screening (*p* = 0.89). PR intervals were not prolonged in any participant, but did increase slightly in both groups between screening and ~ Tmax (mean from 161 to 168 ms in ARV-naïve group (*p* = 0.027) and from 152 to 166 ms in the lopinavir group (*p* = 0.012). However, PR intervals were similar between treatment groups at screening (*p* = 0.41) and ~ Tmax (*p* = 0.62). Lumefantrine concentrations at ~ Tmax were not associated with QTcF (*p* = 0.54) or PR intervals (*p* = 0.12).

### Adverse events

There were 173 adverse events recorded overall, with no serious or severe adverse events. All patients except one in the ARV-naive group experienced at least one adverse event. Within 21 days of starting the full (6-dose) artemether-lumefantrine treatment there were 42 adverse events occurring in the lopinavir group (*n* = 16) and 35 adverse events in the ARV-naïve group (*n* = 18) [Table 5]. Of these adverse events, 27 were considered possibly related to study drug, 15 and 12 in the ARV-naïve and lopinavir groups, respectively (*p* = 0.21). Most events were classified as mild, and all four of the moderate adverse events were not considered related to artemether-lumefantrine. The most common adverse events considered possibly related to artemether-lumefantrine were: headache (*n* = 7, with 4 in ARV-naïve group and 3 in lopinavir group), nausea (*n* = 5, with 3 in ARV-naïve group and 2 in lopinavir group), diarrhoea (*n* = 3, with 1 in ARV-naïve group and 2 in lopinavir group), and flatulence (*n* = 3, all in ARV-naïve group). Among those in the lopinavir group given a single dose of artemether-lumefantrine during Phase 1 (*n* = 18), there were 37 treatment emergent adverse events, seven of which were considered possibly related to artemether-lumefantrine (Additional file 1), with a trend (*p* = 0.095) towards fewer adverse reactions than in those given the full 6-dose artemether-lumefantrine treatment, regardless of the antiretroviral regimen used. A further 11 AEs were reported before the artemether-lumefantrine dose was administered, and another 27 after 21 days of follow up (mostly between the single and multiple artemether-lumefantrine dose phases in the lopinavir group). Another 21 adverse events were detected in a methods sub-study with more intensive enquiry about adverse events using checklists, in depth interviews and focus group discussions only conducted in some participants in the lopinavir group [22].

**Table 5:** Treatment Emergent Adverse Events by treament group, causality and intensity

	ARV naïve group (*n*=18)				Lopinavir group (*n*=16)			
	AL suspected		AL not suspected		AL suspected		AL not suspected	
	Mild	Moderate	Mild	Moderate	Mild	Moderate	Mild	Moderate
Gastrointestinal disorders								
Abdominal pain			1					
Constipation							1	
Decreased appetite	1		1					
Diarrhoea	1		1		2		1	
Dyspepsia	2							
Epigastric discomfort			1		1			
Flatulence	3							
Gastrooesophageal reflux disease							1	
Nausea	3				2			
Vomiting					2			
Infections and infestations								
Gingivitis							1	
Influenza-like illness			3				2	
Nervous system disorders								
Dizziness	1				1			
Headache	4		3	1	3		2	
Presyncope								1
Respiratory, thoracic and mediastinal disorders								
Nasal congestion							1	
Rhinorrhoea							1	
Skin and subcutaneous tissue disorders								
Abscess							1	
Body tinea			1					
Rash			1				1	
Seborrhoeic dermatitis							1	
General disorders and administration site conditions								
Fatigue					1		1	
Injection site reaction			2				3	
Musculoskeletal and connective tissue disorders								
Back / neck pain							3	
Muscle twitching			2					
Renal and urinary disorders								
Frequency of micturition							1	
Urinary tract infection			1				1	
Reproductive system and breast disorders								
Vaginal discharge			1					
Immune system disorders								
Seasonal allergy							1	
Vascular disorders								
Epistaxis							1	
Hypertension			1				1	
Eye disorders								
Uveitis								1
Psychiatric disorders								
Alcoholic hangover								
Self-induced vomiting							1	
Metabolism and nutrition disorders								
Hypercholesterolaemia								
Oedema peripheral							1	
Injury, poisoning and procedural complications								
Skin wounds								1
TOTAL	15	0	19	1	12	0	27	3

## Discussion

We investigated the safety and pharmacokinetics of artemether-lumefantrine after the recommended six-dose regimen in HIV-infected adult patients, comparing results in ARV-naïve patients with those on lopinavir-based antiretroviral therapy. We found that those on antiretroviral therapy had a 10-fold increase in their day-7 lumefantrine concentrations, with an almost five-fold increase in their lumefantrine AUC_(0-inf)_ and almost three-fold increase in the maximum lumefantrine concentration. Despite this substantially elevated exposure, detailed assessments for clinical, haematological, biochemical or electrocardiographic adverse events raised no safety concerns associated with concomitant artemether-lumefantrine and lopinavir-based ART administration. There were no serious adverse events, and most adverse events were mild in intensity. Within 21 days of starting artemether-lumefantrine there were similar numbers of treatment emergent adverse events (42 vs. 35) and adverse reactions (12 vs. 15) in the lopinavir and ARV-naïve groups, respectively.

Lumefantrine is chemically similar to halofantrine, which is known to cause significant QT prolongation and cardiac arrhythmias even at standard doses. This structural similarity initially raised the concern of potential cardiac toxicity, but this was not confirmed in a prospective study [23]. The electrocardiographic assessments in the lopinavir group, including those at the time of predicted maximal lumefantrine concentration, did not show prolonged PR or QTcF intervals, and were not significantly different from the intervals found in the ARV-naïve group. This is consistent with findings by Byakika-Kibwika et al. who assessed cardiac conduction safety in HIV-positive adults, and found that QTc intervals remained well within the normal limits over the 72 h after a *single* artemether-lumefantrine dose, although the mean QTc interval after AL administration was longer in the lopinavir arm compared to the ARV-naïve arm [24].

Although artemether pharmacokinetic parameters were not significantly different between treatment groups in either period, we found a significant dose-occasion effect with five-fold decreases in artemether maximal concentrations, and more than two-fold decreases in AUC between the first and last dose. This is expected given the auto-induction previously described with the artemisinins [16, 25–28]. The dihydroartemisinin maximal concentration and exposure were almost double in the lopinavir group compared to the ARV-naïve group at 0 to 8 h, although these were similar between treatment groups after the last dose. Artemether and dihydroartemisinin exposure in our ARV-naïve group were similar to the results from previously published healthy volunteer studies, suggesting that there is not a marked HIV disease effect [19, 29]. Unlike the findings in a single artemether-lumefantrine dose study in HIV-infected Ugandan adults [30], our dihydroartemisinin concentrations were higher in the lopinavir group at 0–8 hours, which was also reported by German et al. in healthy volunteers [29]. If confirmed, this increased artemisinin exposure with lopinavir/ritonavir may result in some benefit, particularly in the light of confirmed artemisinin resistance having spread from western Cambodia across mainland South East Asia, from southern Vietnam to western Myanmar [31–33].

Two previous studies in American and Ugandan adults have examined the interaction of lopinavir-based antiretroviral therapy and artemether-lumefantrine, using different methods to our study [25, 29]. Due to concerns about substantially elevated lumefantrine concentrations we included a single-dose phase, which then determined the dosing regimen used for our second six-dose phase. Our single-dose phase found a doubling of the lumefantrine exposure, which was similar to the result found in a single-dose study of Ugandan malaria-negative, HIV-infected patients on lopinavir-based antiretroviral therapy, and in a healthy volunteer, six-dose study conducted in the United States (Table 6). In the six-dose phase of our trial, the lumefantrine exposures in our ARV-naïve group were similar to those in American healthy volunteers (445 vs. 456 ug.h/mL respectively). However, our patients on lopinavir-based antiretroviral therapy had 1.6-fold higher maximal concentrations and 2.3-fold higher exposures than the healthy volunteers on lopinavir in the American study. Hoglund et al. (2015) who reanalysed the pharmacokinetic data from the single-dose study in Ugandan HIV-infected, malaria-negative adults [30] using non-linear mixed-effect modelling, also found that lopinavir-ritonavir increased lumefantrine exposure by 439 %, largely explained by its clearance being decreased by 62 %.

**Table 6:** Drug interaction studies between artemether-lumefantrine and lopinavir/ritonavir, showing median lumefantrine, artemether and dihydroartemisinin pharmacokinetic parameters with and without lopinavir/ritonavir

Study (Reference)	Population	Comparator groups	Artemether- lumefantrine dose	Concomitant fat intake	Samples per participant (matrix)	Lumefantrine			Artemether		Dihydro-artemisinin	
						AUC	Cmax	Day 7	AUC	Cmax	AUC	Cmax
Kredo	HIV infected, malaria negative adults	Parallel: Lopinavir based ART vs. ARV-naïve	Single dose (Phase 1)	Yes	22 (Plasma)	Single dose: AUC_(0-inf)_ 1852 vs. 1133 μg.h/mL	5.26 vs. 2.50 μg/mL	NA	NA	NA	NA	NA
			Standard 6-dose regimen (Phase 2)	Yes	33 (Plasma)	AUC_(0-inf)_ 2477 vs. 445 μg.h/mL	28.15 vs 8.76 μg/mL	3170 vs. 336 ng/mL	AUC_(0-8h)_ 220 vs. 151 ng.h/mL	85.8 vs. 59.7 ng/mL	AUC_(0-8h)_ 283.6 vs. 123.8 ng.h/mL	77.5 vs. 42.2 ng/mL
Byakika-Kibwika^25^	HIV infected, malaria negative adults	Parallel: Lopinavir based ART vs. ARV- naïve	Single dose	Yes	9 (Plasma)	AUC_(0-inf)_ 267 vs. 47 μg.h/mL	7.10 vs. 2.53 μg/mL	NA	AUC_(0-inf)_ 162 vs 271 ng.h/mL	56 vs 112 ng/mL	AUC_(0-inf)_ 180 vs 217 ng.h/mL	73 vs 66 ng/mL
German^29^	Healthy adult volunteers	Cross-over: Artemether lumefantrine given before and after 26 days Lopinavir-based ART	Standard 6 dose regimen	Yes	10 (Plasma)	AUC_(0–264)_ 1073 vs 456	17.4 vs 12.5 μg/mL	NA	AUC_(0-inf)_ 40.5 vs. 62.0 ng.h/mL	11.2 vs 14.3 ng/mL	AUC_(0-inf)_ 109 vs 198 ng.h/mL	37.3 vs 58.8 ng/mL
Achan^35^	HIV-infected children with malaria on ART	Parallel: Lopinavir-based ART vs. NRTI-based antiretrovirals	Standard 6 dose regimen	Not reported	1 (Capillary blood)	NA	NA	Lopinavir: 926 ng/mL	NA	NA	NA	NA
								Nevirapine: 388 ng/mL				
								Efavirenz: 97 ng/mL				

To minimise any safety risks, and to obtain an estimate of the effect size of the pharmacokinetic drug-drug interaction without confounding by any malaria disease effect, neither our study nor both studies in adults cited above included malaria patients [25, 29]. Thus, we could not determine whether increased lumefantrine exposure improved antimalarial therapeutic efficacy. Previous studies in malaria patients have shown that the day-7 lumefantrine concentration is the most important single concentration measure in terms of its correlation with the area under the concentration time curve and its association with treatment response [4, 11, 34]. In a large pooled analysis in 2528 patients, treatment failure was associated with low day-7 lumefantrine concentrations; the risk of recrudescence decreased by 36 % (HR 0.64 (0.55 to 0.74) <0.001) and the risk of reinfection decreased by 21 % (HR 0.79 (0.72 to 0.87) <0.001) with a doubling of lumefantrine concentrations [11]. Thus the marked increases in lumefantrine concentration observed in our lopinavir group would be expected to reduce their risk of treatment failure. This has been evaluated in malaria and HIV co-infected children, but not yet in co-infected adults [35, 36]. In co-infected Ugandan children under six-years of age (median age 2.9 years), recurrent malaria and malaria incidence were lower following artemether-lumefantrine treatment in those on lopinavir-based ART than in those on non-nucleoside reverse transcriptase inhibitor (NNRTI)-based ART. The Ugandan paediatric lopinavir group had elevated day-7 lumefantrine concentrations of 388 ng/mL and day-7 concentrations above 300 ng/mL were associated with a significantly decreased risk of malaria recurrence within 63 days.

Previous studies show that the absorption of lumefantrine was close to saturated at the currently recommended dose [37], which is a major obstacle for optimising dosage recommendations for patient sub-groups who do not achieve target concentrations with the currently recommended dosage regimens [12]. However, we showed that an increase in lumefantrine mg/kg dose was associated with a significant increase in the lumefantrine Cmax and AUC_(0-inf)_, in the lopinavir group but not the ARV-naïve group. Lumefantrine is N-debutylated by CYP3A4, but desbutyl-lumefantrine represents approximately 0.3–1 % of the parent exposure [12] suggesting inhibition of intestinal CYP3A4 and possibly other transporters as a mechanism. These findings may contribute towards a better understanding of the mechanism underlying the non-linear relationship between lumefantrine dose and bioavailability, and of interventions that could be studied in key target populations in whom lumefantrine is currently sub-optimally dosed.

## Conclusions

Despite markedly higher lumefantrine exposure, intensive monitoring in our relatively small study raised no safety concerns associated with using the current recommended six-dose regimen of artemether-lumefantrine in HIV-infected adult patients receiving lopinavir-based antiretroviral therapy. Elevated lumefantrine concentrations have been shown to reduce the risk of treatment failure as reported previously in malaria patients of all ages [4, 11], and in malaria and HIV co-infected children [35, 36]. Further evidence in adults co-infected with malaria and HIV is required to substantiate these results. Our ARV-naïve patients confirmed previous studies’ findings that lumefantrine absorption is close to saturation with currently recommended doses, but this dose-limited absorption was overcome in those on lopinavir-based ART.

## Additional file

Additional file 1:
**Treatment Emergent Adverse Events within 21 days after a single artemether-lumefantrine dose in 18 HIV-infected adults on lopinavir-based antiretrovirals, by causality and intensity.** (DOCX 13 kb)
