# Supplementary material for: The interaction between artemether-lumefantrine and lopinavir/ritonavir-based antiretroviral therapy in HIV-1 infected patients
Source: BMC Infect Dis. 2016 Jan 27;16:30. doi: 10.1186/s12879-016-1345-1 (PMC4728832; doi:10.1186/s12879-016-1345-1)
Supplement: Additional file 1: — Treatment Emergent Adverse Events within 21 days after a single artemether-lumefantrine dose in 18 HIV-infected adults on lopinavir-based antiretrovirals, by causality and intensity. (DOCX 13 kb) [file 12879_2016_1345_MOESM1_ESM.docx]

Additional File 1: Treatment Emergent Adverse Events by treatment group, causality and intensity

| MedDRA preferred term | Single Artemether-lumefantrine dose (n=18) | | | |
| --- | --- | --- | --- | --- |
|  | AL suspected | | AL not suspected | |
|  | Mild | Moderate | Mild | Moderate |
| **Gastrointestinal disorders** | | | | |
| Constipation | 1 | 1 |  |  |
| Decreased appetite |  |  | 1 |  |
| Diarrhoea | 3 |  | 1 |  |
| Heartburn |  |  | 1 |  |
| Nausea |  |  | 1 |  |
| Oral pruritus |  |  | 1 |  |
| Vomiting |  |  | 1 |  |
| **Infections and infestations** | | | | |
| Conjunctivitis |  |  | 1 |  |
| Herpes simplex |  |  | 1 |  |
| Influenza-like illness |  |  | 3 |  |
| **Nervous system disorders** | | | | |
| Headache | 2 |  | 2 |  |
| **Respiratory, thoracic and mediastinal disorders** | | | | |
| Bronchitis |  |  | 2 |  |
| Cough |  |  | 1 |  |
| Laryngitis |  |  | 1 |  |
| Upper respiratory tract infection |  |  | 2 |  |
| **Skin and subcutaneous tissue disorders** | | | | |
| Boils |  |  | 1 |  |
| Folliculitis |  |  | 1 |  |
| **General disorders and administration site conditions** | | | | |
| Injection site reaction |  |  | 1 |  |
| Pyrexia |  |  | 1 |  |
| **Musculoskeletal and connective tissue disorders** | | | | |
| Back / neck pain |  |  | 2 |  |
| **Reproductive system and breast disorders** | | | | |
| Urogenital trichomoniasis |  |  | 1 |  |
| **Immune system disorders** | | | | |
| Conjunctivitis allergic |  |  | 1 |  |
| **Metabolism and nutrition disorders** | | | | |
| Hypercholesterolaemia |  |  | 2 |  |
| **Blood and lymphatic system disorders** | | | | |
| Iron deficiency anaemia |  |  |  | 1 |
| **TOTAL** | 6 | 1 | 29 | 1 |
